# *Entamoeba gingivalis* in Acute Osteomyelitis of the Mandible

**DOI:** 10.1155/2011/357301

**Published:** 2011-07-17

**Authors:** Feriyl Bhaijee, Diana Bell

**Affiliations:** ^1^University of Mississippi Medical Center, 2500 North State Street, Jackson, MS 39216, USA; ^2^Department of Pathology, MD Anderson Cancer Center, 1515 Holcombe Boulevard, Houston, TX 77030, USA

## Abstract

An 86-year-old woman presented with osteonecrosis of the mandible following bisphosphonate therapy for multiple myeloma, and underwent surgical debridement and multiple dental extractions. Histopathologic examination of the necrotic bone fragments revealed acute osteomyelitis with mixed flora and organisms morphologically consistent with *Entamoeba gingivalis*. In addition to oral scrapings and sputum, *E. gingivalis* has been identified in specimens obtained from the uterus, cervix, neck lymph nodes, and lung. It is rarely found in lesions of the head and neck. We present an unusual case of *E. gingivalis* in acute osteomyelitis of the mandible, following bisphosphonate therapy for multiple myeloma. To our knowledge, this is the first reported case of *E. gingivalis* in association with osteomyelitis.

## 1. Introduction

*Entamoeba gingivalis* is one of seven *Entamoeba* species that commonly infect humans and is usually found in the oropharynx, where it is considered a commensal organism. *E. gingivalis* is more common in patients with poor dentition, periodontal disease, or immune suppression. 

Herein, we present an unusual case of *E. gingivalis* associated with acute osteomyelitis of the mandible.

## 2. Case Presentation

An 86-year-old woman with a background history of hypertension, multiple myeloma, hypothyroidism, and osteoarthritis, presented to the Dental Oncology service with osteonecrosis of the right mandible, secondary to bisphosphonate treatment. She also had poor dentition, multiple abscesses, advanced periodontal disease, and gross dental caries. Her past surgical history included a right mastectomy for carcinoma and an open reduction and internal fixation of her right humerus following a fracture. The patient did not smoke tobacco or consume alcohol, and her family history was noncontributory. Surgical debridement of the right mandible was recommended in order to prevent septicaemia and prolonged hospitalization during the course of her chemotherapy, as well as prevent progression of the osteonecrosis. 

Prior to surgery, her white cell count was 7.9 × 10^3^/mm^3^, with no evidence of systemic infection. She underwent multiple dental extractions, two quadrant alveoplasty, curettage of abscesses, and excision of necrotic bone from her right mandible. There were no postoperative complications.

### 2.1. Pathology

**Figure 1:**
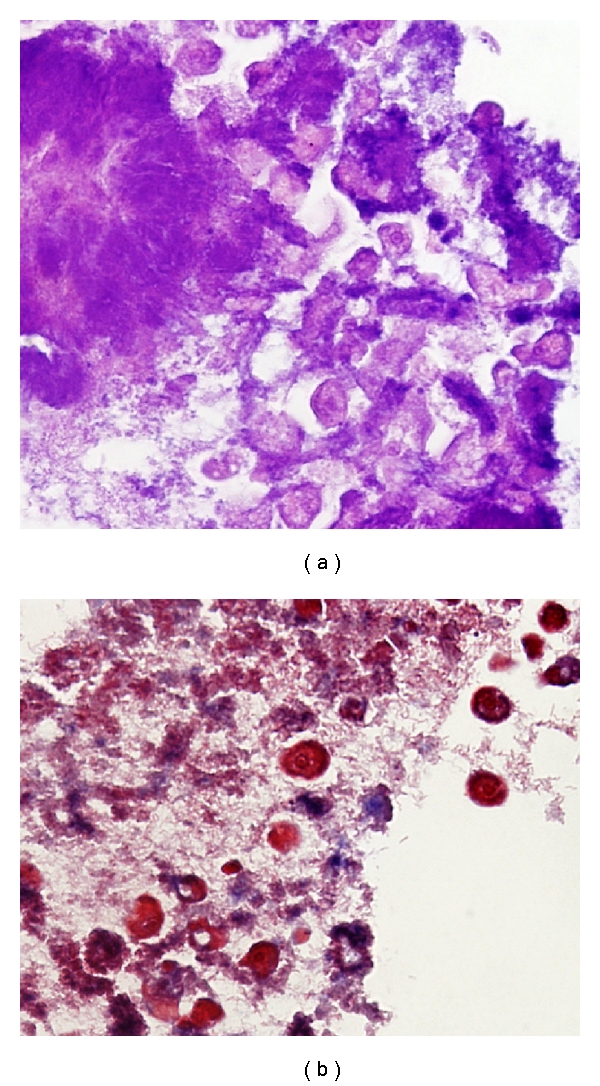
*Entamoeba gingivalis.* (a) Acute osteomyelitis with mixed flora, including branching rods and cocci, colonies of filamentous bacteria consistent with *Actinomyces*, and numerous organisms morphologically consistent with *Entamoeba gingivalis*; (b) Trichrome staining highlights the forms morphologically consistent with *Entamoeba gingivalis. *

A 2.0 cm fragment of dark brown necrotic bone was decalcified and submitted for histologic processing. Microscopic examination revealed acute osteomyelitis with mixed flora, including branching rods and cocci, colonies of filamentous bacteria consistent with *Actinomyces*, and numerous organisms morphologically consistent with *Entamoeba gingivalis* (Figure 1(a)). The organisms had small central vesicular nuclei, peripheral clumped chromatin, finely granular cytoplasm, and multidirectional pseudopods.

Gomori methenamine silver (GMS), periodic acid-Schiff (PAS), and trichrome stains highlighted the abundant bacterial forms (rods and cocci). PAS and trichrome stains highlighted the forms morphologically consistent with *Entamoeba gingivalis* (Figure 1(b)), which were negative for CD68 and CD163 immunohistochemical stains.

## 3. Discussion

An 86-year-old woman presented with osteonecrosis of the mandible following bisphosphonate therapy for multiple myeloma, and underwent surgical debridement and multiple dental extractions. Histopathologic examination of the necrotic bone fragments revealed acute osteomyelitis with mixed flora and organisms morphologically consistent with *Entamoeba gingivalis*. 

*Entamoeba gingivalis* is one of seven *Entamoeba* species that infect humans, including *E. histolytica*, which causes amoebic dysentery and amoebic liver abscesses. Both protozoa, *E. gingivalis* and *E. Histolytica,* are commonly found in cytologic and histopathologic specimens. Diagnostic distinction is important and may have therapeutic implications: while *E. gingivalis* is susceptible to metronidazole [1], *E. histolytica* infection may require additional therapy, such as paromomycin, to eradicate intestinal luminal cysts. *Entamoeba* trophozoites contain a single nucleus and a variable number of pseudopods, which form clear bulges visible on light microscopy. The trophozoite feeds on bacteria and divides by simple binary fission to form two small daughter cells. With the exception of *E. Gingivalis *[2], almost all *Entamoeba* species form cysts during the transmission stage; variation in size and nuclei aid in species identification. *E. gingivalis* is transmitted almost entirely through oral contact [2]. As seen in our case, colonies morphologically resembling *Actinomyces* are often associated with *E. gingivalis*.

*E. gingivalis* has a small but prominent central nucleus (karyosome) with a peripheral rim of clumped chromatin (characteristic of the genus) and finely granular cytoplasm [3]. Morphologically, the trophozoites of *E. gingivalis* resemble those of *E. histolytica,* although the former are usually larger (10–35 *μ*m versus 15–20 *μ*m). Other features of *E. gingivalis* can be used to distinguish it from *E. Histolytica *[4]; for example, unlike *E. histolytica*, *E. gingivalis* has no cyst stage in vivo. The contents of the intracytoplasmic food vacuoles differ between species: the trophozoites of *E. histolytica* may contain erythrocytes in invasive disease, while those of *E. gingivalis* contain basophilic fragments of leukocytic nuclei and cellular debris. Though the nuclei of these amoebae are very similar in appearance, subtle differences may be apparent: the trophozoites of *E. gingivalis* have a coarse karyosome, coarse peripheral chromatin, and multidirectional pseudopods, while those of *E. histolytica* have a fine central karyosome, delicate peripheral chromatin, and a single unidirectional pseudopod. As in our case, *E. gingivalis* is often seen in the background of marked inflammation with abundant neutrophils.

Most *Entamoeba* species reside in hosts' intestines, with the exception of *E. gingivalis*, which inhabits the oropharynx and is thought to be a commensal organism in humans. *E. gingivalis* is more common in patients with poor dentition, oral hygiene, or with immune suppression. In the largest series to date, Sefer et al. report *E. gingivalis* in 135 patients with various oral pathologies, including dental caries, parodontopathies, pulpitis, gangrene, and ulceronecrotic stomatitis [5]. A recent study of the prevalence of *E. gingivalis* infection among 551 college students in Tangshan, China, demonstrated an overall prevalence of 28.3%; higher prevalence rates were observed among students with oral disease, poor oral hygiene, and those who did not regularly chew xylitol gum [6]. Lucht et al. found that among 13 HIV-infected Swedish patients with periodontal disease, 10 (77%) had *E. gingivalis* in their saliva or dental plaque [7]. Moreover, *E. gingivalis* was the only protozoa identified in this cohort, and its presence was *not* related to the degree of immunodeficiency. 

In addition to oral scrapings and sputum [8, 9], *E. gingivalis* has been identified in specimens obtained from the uterus, cervix [9, 10], neck lymph nodes, and lung [4]. *E. gingivalis* is rarely found in lesions of the head and neck or in association with osteomyelitis. A search of the existing medical literature revealed a single case of *E. gingivalis* in a neck nodule [11] following radiotherapy, and no reported cases of osteomyelitis, as seen in our case. Moreover, it is important to note that most reports in the existing literature are based on the morphologic identification of *E. gingivalis,* not molecular or culture methods of diagnosis.

Using light microscopy, *E. gingivalis* has been identified in diseased gingival pockets for nearly a century. Identification of the protozoa on H&E stains may be difficult. Special stains, such as Gomori-Wheatley trichrome, iron hematoxylin, and PAS, stain both *E. gingivalis* and *E. histolytica*, but these are nonspecific [9]. Fluorescein-labelled anti-*E. histolytica* serum may distinguish the protozoa in stool specimens [9], as do highly specific molecular techniques. Trim et al. have recently developed a novel molecular biology approach to detect *E. gingivalis* in gingival pockets [12]. A conventional PCR (which used a previously described primer set specific for the small subunit ribosomal RNA gene (SSU rDNA) of *E. gingivalis*) was compared with a new real-time PCR assay (which used a primer set designed to amplify a 135-bp fragment inside the SSU rDNA of *E. gingivalis*). The former detected *E. gingivalis* in 27% of diseased gingival pockets, while the latter detected the protozoa in 69% of diseased pockets. Interestingly, neither of the PCR assays detected *E. gingivalis* in any healthy gingival pockets. Trim et al. conclude that *E. gingivalis* is only associated with diseased gingival pocket sites and that the newly described methodology may provide a novel eukaryotic cell marker of disease status in gingival pockets. 

In conclusion, we present an unusual case of *E. gingivalis* in acute osteomyelitis of the mandible, following bisphosphonate therapy for multiple myeloma. To our knowledge, this is the first reported case of *E. gingivalis* in association with osteomyelitis.

## References

[1] Muller M (1983). Mode of action of metronidazole on anaerobic bacteria and protozoa. *Surgery*.

[2] Wantland WW, Wantland EM, Winquist DL (1963). Collection, identification, and cultivation of oral protozoa. *Journal of Dental Research*.

[3] Okada H, Matsumato T, Miyuki K, Nakahira T, Omura M, Yamamoto H (2002). Clinico pathological and cytolological study of *Entamoeba* gingivalis. *The Journal of the Japanese Society of Clinical Cytology*.

[4] Jian B, Kolansky A, Baloach Z, Gupta PK (2008). Entamoeba gingivalis pulmonary abscess-diagnosed by fine needle aspiration. *CytoJournal*.

[5] Sefer M, Boanchis AL, Chaouki SH, Ganescu V, Constantin P (1989). Periodontal diseases with Entamoeba gingivalis. *Revista de Chirurgie, Oncologie, Radiologie, O R L, Oftalmologie, Stomatologie*.

[6] Huang W, Shi JL, Li CL (2009). *Entamoeba* gingivalis infection among college students in Tangshan. *Zhongguo ji sheng chong xue yu ji sheng chong bing za zhi*.

[7] Lucht E, Evengard B, Skott J, Pehrson P, Nord CE (1998). Entamoeba gingivalis in human immunodeficiency virus type 1-infected patients with periodontal disease. *Clinical Infectious Diseases*.

[8] Sutliff WD, Green FD, Suter LS (1951). Entamoeba gingivalis in pulmonary suppuration. *The American Journal of Tropical Medicine and Hygiene*.

[9] Gupta PK (1982). Intrauterine contraceptive devices. Vaginal cytology, pathologic changes and clinical implications. *Acta Cytologica*.

[10] Ruehsen MD, McNeill RE, Frost JK, Gupta PK, Diamond LS, Honigberg BM (1980). Ameba resembling *Entamoeba* gingivalis in the genital tract of IUD users. *Acta Cytologica*.

[11] Perez-Jaffe L, Katz R, Gupta PK (1998). *Entamoeba* gingivalis identified in a left upper neck nodule by fine- needle aspiration: A case report. *Diagnostic Cytopathology*.

[12] Trim RD, Skinner MA, Farone MB, Dubois JD, Newsome AL Use of PCR to detect Entamoeba gingivalis in diseased gingival pockets and demonstrate its absence in healthy gingival sites.

